# *BANF1* Is Downregulated by IRF1-Regulated MicroRNA-203 in Cervical Cancer

**DOI:** 10.1371/journal.pone.0117035

**Published:** 2015-02-06

**Authors:** Langyong Mao, Yan Zhang, Wenjuan Mo, Yao Yu, Hong Lu

**Affiliations:** 1 State Key Laboratory of Genetic Engineering, School of Life Sciences, Fudan University, Shanghai, China; 2 Department of Gynecology and Obstetrics, Changhai Hospital, Shanghai, China; 3 Shanghai Engineering Research Center of Industrial Microorganisms, Shanghai, China; 4 Shanghai Collaborative Innovation Center for Biomanufacturing Technology, Shanghai, China; The University of Tennessee Health Science Center, UNITED STATES

## Abstract

MicroRNAs (miRNAs) play important roles in various biological processes and are closely associated with the development of cancer. In fact, aberrant expression of miRNAs has been implicated in numerous cancers. In cervical cancer, miR-203 levels are decreased, although the cause of this aberrant expression remains unclear. In this study, we investigate the molecular mechanisms regulating miR-203 gene transcription. We identify the miR-203 transcription start site by 5’ rapid amplification of cDNA ends and subsequently identify the miR-203 promoter region. Promoter analysis revealed that IRF1, a transcription factor, regulates miR-203 transcription by binding to the miR-203 promoter. We also demonstrate that miR-203 targets the 3’ untranslated region of *BANF1*, thus downregulating its expression, whereas miR-203 expression is driven by IRF1. MiR-203 is involved in cell cycle regulation and overexpression of miR-203 suppresses cervical cancer cell proliferation, colony formation, migration and invasion. The inhibitory effect of miR-203 on the cancer cells is partially mediated by downregulating its target, *BANF1*, since knockdown of *BANF1* also suppresses colony formation, migration and invasion.

## Introduction

It is estimated that approximately 30% of human genes are regulated by miRNAs. The deregulation of miRNAs can alter the expression levels of target genes, resulting in abnormal cellular processes [1]. Recently, multiple studies have shown that aberrant expression of microRNAs (miRNAs) is implicated in numerous disease states and that this expression is closely associated with the development of human malignancies [2]. In addition, some miRNAs have been explored as potential biomarkers for the diagnosis or prognosis of human diseases [3–6]. Therefore, the mechanisms involved in miRNA deregulation are a matter of urgent and important research.

The mechanisms leading to aberrant expression of miRNAs are not yet completely understood. miRNAs are typically transcribed by RNA polymerase II into primary miRNAs (pri-miRNAs), which are processed by Drosha to pre-miRNAs and then further cleaved by Dicer to short, mature miRNAs [7,8]. Theoretically, aberrant expression of miRNAs can be caused by various mechanisms, including deletions, amplifications and mutations involving miRNA loci, epigenetic modifications and the dysregulation of transcription factors that target specific miRNAs. At the genomic level, chromosomal abnormalities and epigenetic modifications, including mutations, CpG island methylation and repressive histone modifications, are responsible for miRNA silencing in cancer. At the transcriptional level, miRNAs interact with transcription factors and transcription inhibitors to create a dynamic balance that regulates their expression [1].

Cervical cancer is one of the most commonly diagnosed cancers and the leading cause of cancer deaths in females worldwide, accounting for 9% of new cancer cases and 8% of total cancer deaths among females [9]. Aberrant miRNA expression has been found in cervical cancer [10–12], and a large number of aberrant miRNA functions have been reported globally [13]. However, most studies have focused on the aberrant expression of miRNA, while the mechanisms involved in miRNA deregulation are less reported.

MiR-203 has been reported to be dysregulated and to function as a tumor suppressor in hepatocellular carcinoma, prostate cancer, lung cancer, esophageal cancer, and cervical cancer [14–18]; however, the mechanism leading to the aberrant expression of miR-203 is not completely clear. In this study, we identify the miR-203 transcription start site (TSS) by 5’ rapid amplification of cDNA ends (5’ RACE) and subsequently identify the miR-203 promoter sequence. We demonstrate that miR-203 targets the 3’ untranslated region (3’UTR) of *BANF1*, thus downregulating *BANF1* expression, and that miR-203 expression is driven by the transcription factor IRF1. Our study establishes a linear signaling pathway from *IRF1* to *BANF1* that may contribute to *BANF1*-induced tumorigenesis in cervical cancer. This work contributes to the comprehensive understanding of the mechanism of HPV-mediated cervical cancer progression.

## Materials and Methods

### Ethics statement

Human cervical cancer tissues and adjacent normal cervical tissues were collected from Shanghai Changhai Hospital (Shanghai, China). All patients provided written consent, and the study was approved by the Ethical Committees of Shanghai Changhai Hospital and Fudan University (reference number 2011-120).

### Cell culture, RNA molecules and transfection

Cervical cancer cell lines (CaSki and HeLa) were obtained from the American Type Culture Collection (ATCC) and cultured in Dulbecco’s Modified Eagle Medium (DMEM, Gibco, USA) containing 10% (v/v) fetal bovine serum (FBS, Gibco) at 37°C in a 5% CO_2_ atmosphere. Synthetic miRNA molecules, including the miR-203 mimic, inhibitor and negative control, were purchased from Ambion, USA. The small interfering RNAs against *BANF1* and negative control were synthesized by Genepharma (Shanghai, China) [19]. The sequences were as follows: siRNA-BANF1, 5’-CCAGGUGCAUUUAAAGAAATT-3’ and control sequence, 5’-UUUCUUUAAAUGCACCUGGTT-3’. Cells were transfected using Lipofectamine 2000 (Invitrogen, USA) according to the manufacturer’s instructions.

### RNA extraction and quantitative real time PCR (qRT-PCR)

Total RNA was extracted from tissues and cells using TRIzol reagent (Ambion, USA) and reverse transcribed using the PrimeScript RT reagent Kit (TaKaRa, China) according to the manufacturer’s instructions. The primers used are listed in S1 Table. For miR-203 detection, RNA was reverse transcribed by a specific reverse-transcription primer (RT-miR-203). For mRNA detection, RNA was reverse transcribed by Random 6 mers and Oligo dT Primer. SYBR Premix Ex Taq (TaKaRa, China) was used to quantitate mature miR-203 and mRNA expression using the LightCycler 480 II Real Time PCR system (Roche, Germany). RNU6B and glyceraldehyde-3-phosphate dehydrogenase (GAPDH) were used as internal controls for miR-203 and mRNA expression, respectively. Relative gene expression levels were determined using the delta Ct method [20] and expressed as the average of three independent experiments ± the standard deviation.

### 5’ rapid amplification of cDNA ends (5’RACE)

The TSS of the miR-203 primary transcript (from HeLa cells) was determined using a 5'-Full RACE kit (TaKaRa, China). Two micrograms of RNA was used in each reaction, and HL60 total RNA, which was provided in the kit, was used to analyze the 5’ end of the human *Prohibitin* (PHB) gene (PCR product 750 bp), which served as a positive control. The levels of the miR-203 primary transcript were determined by nested PCR using outer and inner primers (S1 Table). All reactions were performed using the same parameters as suggested in the manual. The PCR products were separated by 1% agarose gel electrophoresis, detected, purified and then sequenced.

### Dual-luciferase reporter assays and transcription factor analysis

The 3’UTR of *BANF1* was amplified from human genomic DNA of HeLa cells and cloned into the psiCHECK-2 reporter vector (Promega, USA), and mutation and deletion of the *BANF1* 3’UTR was achieved by SOE PCR (gene splicing by overlap extension PCR). HeLa cells were co-transfected with the reporter vectors and either miR-203 mimic (30 nM) or miRNA mimic negative control (NC, 30 nM). Luciferase activity was measured using the Dual-Luciferase Reporter Assay System (Promega, USA), and the results were expressed as the normalized ratio of Renilla to firefly luciferase.

For the miR-203 promoter reporter assay, the 744-bp genomic sequence upstream of miR-203 was amplified by PCR and cloned into the pGL3-Basic (Promega, USA) reporter vector. HeLa cells were co-transfected with the promoter reporter vector and either pCDNA-IRF1 or the control plasmid, pcDNA3.1. A Renilla luciferase plasmid, pRL-TK, was used to normalize the transfection efficiency. Cells were lysed at 24 or 48 h after transfection, and luciferase activity was measured with the Dual-Luciferase Reporter Assay System (Promega, USA). The results are expressed as the normalized ratio of firefly to Renilla luciferase and are presented as the means ± SDs from three independent experiments.

### Western blotting

Cells were lysed in RIPA buffer plus protease inhibitors (Millipore, USA). The lysates were then subjected to SDS-PAGE, electrotransferred to PVDF membranes (Millipore, USA), blocked with 5% nonfat milk, incubated with antibodies for 2 hours at room temperature, including anti-beta-tubulin (Abmart, M20005, 1:2000, China), anti-BANF1 (Santa Cruz, sc-33787, 1:200, USA), HRP anti-rabbit (KPL, 4751-1516, 1:2000, USA) and HRP anti-mouse (KPL, 0751-1809, 1:2000, USA), and visualized using cECL Detection Reagents (CWBIO, China). Images were acquired using the Gene Gnome Bio Imaging system (Syngene, UK).

### Chromatin immunoprecipitation (ChIP) assays

ChIP assays were performed using the EZ-Magna ChIP One-Day Chromatin Immunoprecipitation Kit (Millipore, USA) according to the instruction manual. Briefly, approximately 1x10^7^ HeLa cells were cross-linked and lysed by lysis buffer containing Protease Inhibitor Cocktail II. Chromatin was sheared by sonication (high power, 10 cycles of 30 s ‘on’ and 30 s ‘off’; Bioruptor UCD-300, USA) to an average size of 200 to 1000 bp, and the sheared chromatin samples were divided into 50-μl aliquots. Each aliquot of chromatin (1x10^6^ cell equivalents of chromatin) was diluted in 450 μl of dilution buffer. Five microliters (1%) of the supernatant was removed as ‘Input’, and the immunoprecipitating antibody, IRF1 (Abcam, ab26109, 5 μg, UK), and 20 μl of protein A magnetic beads were added to the remaining supernatant. Finally, the immune complexes were collected, washed and eluted, and the cross-links were then reversed. DNA was recovered and analyzed by qRT-PCR. As a negative control, normal mouse IgG was used for immunoprecipitation.

### Cell proliferation and colony formation assays

For the cell proliferation assays, CaSki and HeLa cells were seeded in 96-well plates and transfected with either 30 nM miR-203 mimic or negative control and maintained in full-growth media for 7 days. Cell viability was assessed using an MTT assay. For this assay, cells were seeded in triplicate at the same initial density, and the absorbance at 490 nm was read on sequential days using a plate reader (BioTek, USA). For colony formation assays, 200 cells were seeded in 24-well plates and incubated at 37°C for either 10 days (HeLa cells) or 15 days (CaSki cells). The cells were stained with 0.1% crystal violet and counted if the colony contained more than 50 cells. Experiments were performed in triplicate.

### *In vitro* migration and invasion assays

Invasion and migration assays were performed using 24-well transwell chambers (8 μm pore size polycarbonate membrane, Corning, USA) with or without 150 μg Matrigel (BD, USA). For the migration assays, 5×10^4^ cells in serum-free medium were plated in the upper chamber. For the invasion assays, 1×10^5^ cells in serum-free medium were plated in the upper chamber. Medium with 10% FBS was added to the bottom wells of the chambers. After 20 hours (HeLa cells) or 26 hours (CaSki cells) of incubation, cells adhering to the lower membrane were fixed with methanol and stained with 0.1% crystal violet. The migrated or invaded cells were counted and imaged using an IX51 inverted microscope (Olympus, Japan).

### Flow cytometry assays

Cells were transfected with 30 nM miRNA mimic in 6-well plates, the media was changed the next day, and the cells were incubated at 37°C for another 48 h following transfection. For cell-cycle analysis, cells were resuspended in NP40/PI staining solution (0.01 mg/ml propidium iodide, 0.1% Na citrate, 0.0564% NaCl, 0.025 mg/ml ribonuclease A, 0.3% NP-40) and incubated at 37°C for 30 min. The cells were then analyzed for DNA content using a FACSCalibur Flow Cytometer (BD, USA). Cell-cycle modeling was performed using the Modfit software.

### Statistical analysis

Student’s t-test was used to evaluate significant differences between two groups of data in all appropriate experiments. A *P* value <0.05 was considered significant.

## Results

### Identification of the miR-203 promoter

**Fig 1:**
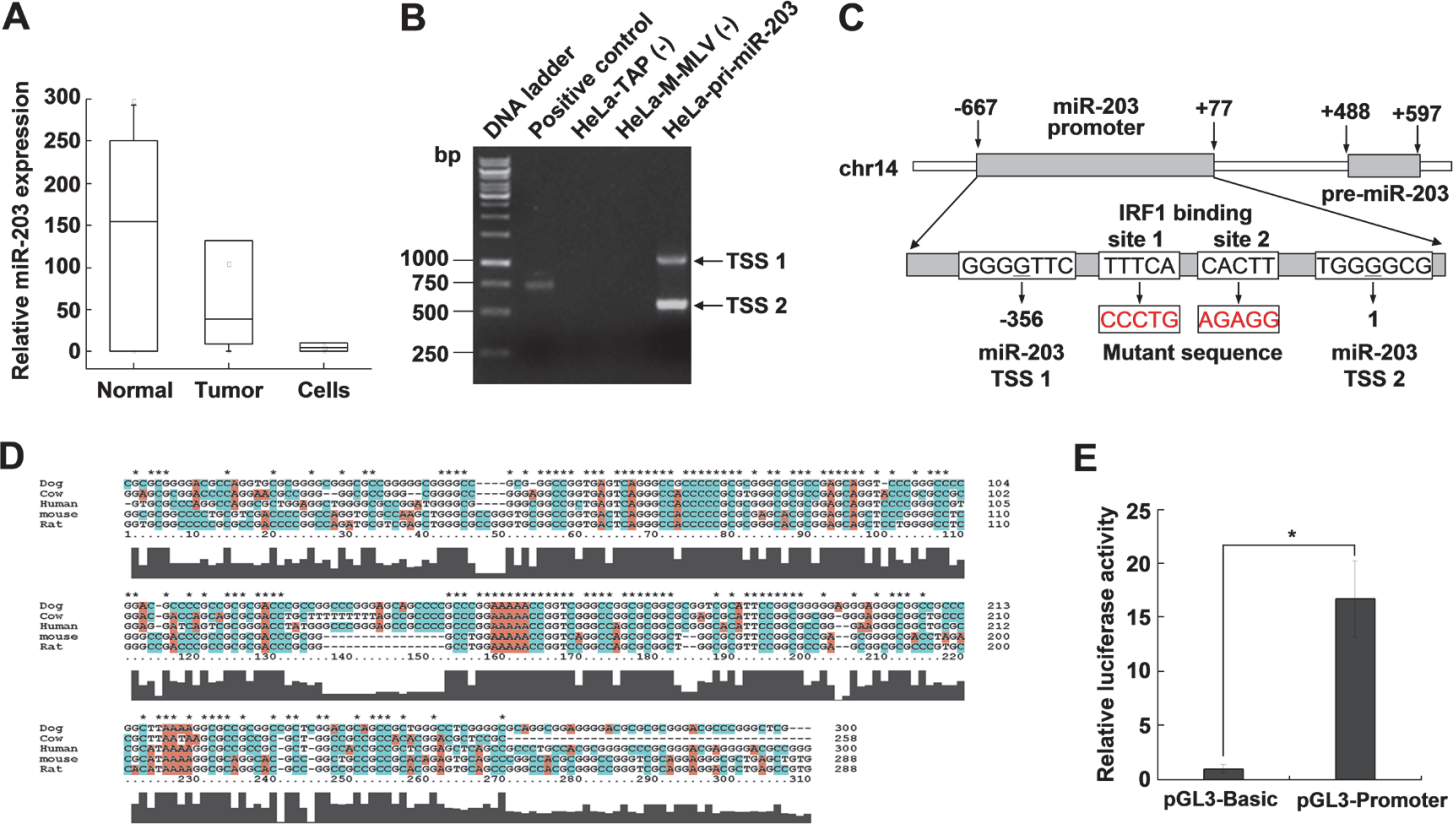
Position of the miR-203 promoter and transcription factor binding sites in the genome. (A) MiR-203 expression levels in normal human cervical tissues, cervical cancer tissues and cervical cancer cell lines (CaSki and HeLa) were measured by qRT-PCR using RNU6B as an internal control. (B) 5’RACE PCR results show the transcription start sites of human (HeLa cells) pri-miR-203. (C) Schematic of the predicted binding sites of IRF1 in the miR-203 gene promoter. The number indicates the relative distance from the 5’ end of the primary miR-203 transcript (indicated as +1). (D) Alignment results show the conservation of miR-203 promoter sequences between various animals. (E) MiR-203 promoter activity analysis by dual-luciferase assays. The predicted miR-203 promoter was cloned into the pGL3-basic vector upstream of the firefly luciferase gene. Firefly luciferase activity was normalized to Renilla luciferase activity and plotted relative to the control (*P<0.05). The results are representative of three independent experiments.

To determine whether miR-203 expression is abnormal in cervical cancer, qRT-PCR was performed on 20 paired patient samples and cervical cancer cell lines (HPV 16+ CaSki and HPV 18+ HeLa). As shown in Fig. 1A, miR-203 expression was substantially downregulated in cervical cancer tissues and cell lines comparing with the adjacent normal cervical tissues. However, the mechanism of this downregulation remains unclear. To explore the regulation of miR-203 expression at the transcriptional level, we first determined the TSS of miR-203 using 5'RACE analysis. As shown in Fig. 1B, there were two 5'RACE PCR products for pri-miR-203, which suggested that miR-203 might have at least two TSSs. The TSS near pre-miR-203 was consistent with that shown in a previous publication [21] and was labeled +1.

We then performed in silico analysis of the miR-203 promoter region (including 2 kb upstream of pre-miR-203) using the online tools FirstEF, PromoterScan, softberry-fprom, and Promoter 2.0 Prediction. These tools predicted that a 1-kb, GC-rich sequence (approximately 70%) near the miR-203 TSS was the promoter (Fig. 1C). Typically, promoter sequences in different species are conserved. Therefore, we aligned the predicted human promoter sequence to the region upstream of pre-miR-203 of mouse, rat, dog and cow and found a highly conserved region (Fig. 1D). We hypothesized that this conserved sequence was most likely the promoter sequence of miR-203. Then, we analyzed the activity of this promoter using luciferase reporter assays. As shown in Fig. 1E, compared with the control vector, the predicted promoter sequence significantly increased the luciferase activity (approximately 17-fold), indicating that this conserved sequence has strong promoter activity.

### IRF1 is involved in the transcriptional regulation of miR-203

**Fig 2:**
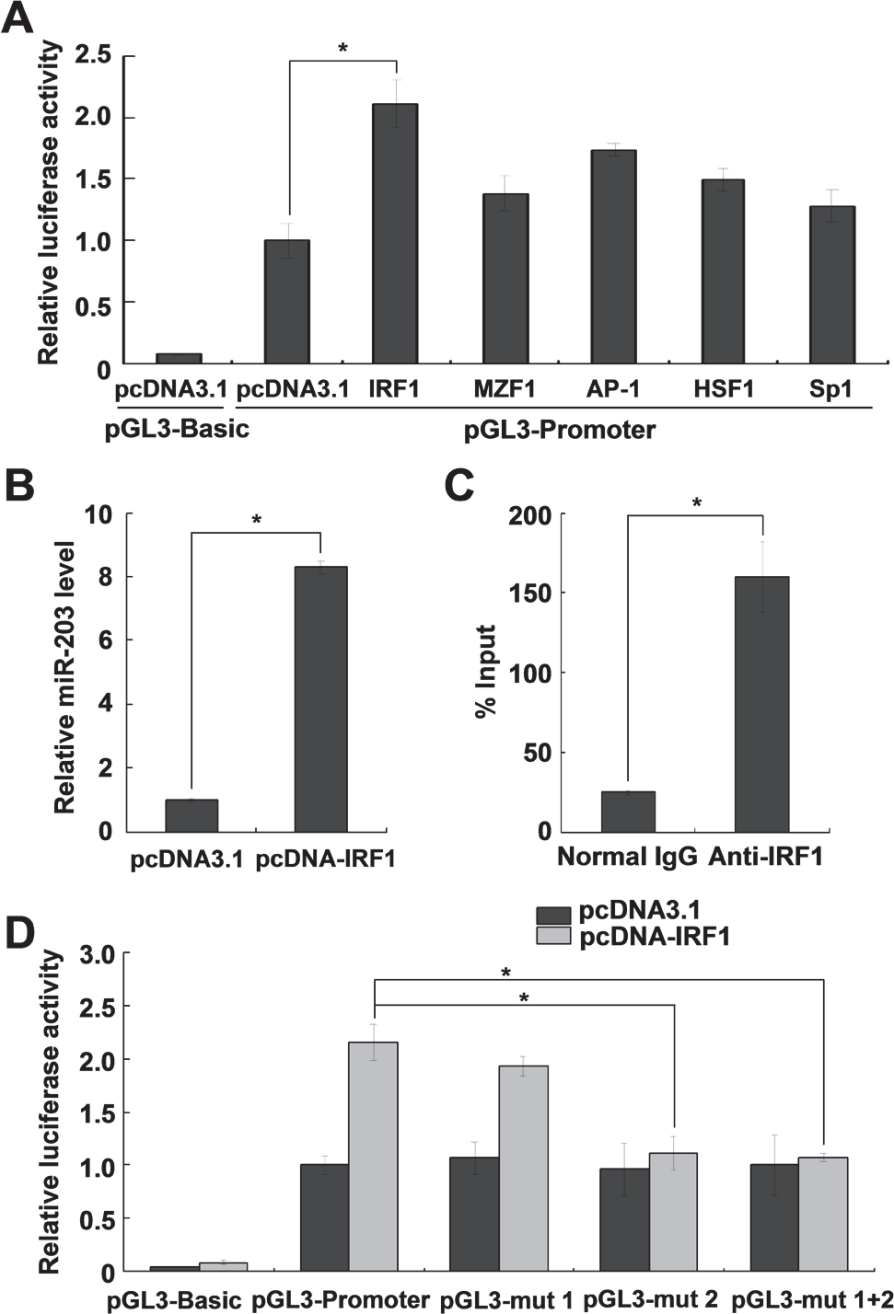
IRF1 is involved in the transcriptional regulation of miR-203. (A) Dual-luciferase assays show the effect of several transcription factors (IRF1, MZF1, AP-1, HSF1, and Sp1) on miR-203 promoter activity. The constructs were co-transfected into HeLa cells with vectors expressing transcription factors and the pRL-TK vector as a transfection control. The wild type pCDNA3.1 plasmid served as a negative control. The relative luciferase activities are shown as the ratio of firefly/Renilla luciferase activity. (B) QRT-PCR assays show the effects of IRF1 overexpression on miR-203 expression. (C) Chromatin immunoprecipitation assays show the *in vivo* interaction between IRF1 and the miR-203 promoter. HeLa cell chromatin fragments were immunoprecipitated with an antibody for IRF1 or negative control antibody (normal IgG). The DNA samples from immunoprecipitates were analyzed by qRT-PCR using primers specific for the miR-203 promoter. (D) Luciferase assays using the mutant promoter constructs. The results are representative of three independent experiments. *P<0.05.

By scanning the identified miR-203 promoter with TRANSFAC (http://www.biobase-international.com/product/transcription-factor-binding-sites), several potential transcription factors (AP-1, HSF1, MZF1, IRF1, and Sp1) were predicted. To validate this prediction, we examined the effects of several potential transcription factors on miR-203 promoter-driven luciferase activity. Among these predicted transcription factors, overexpression of IRF1 in HeLa cells significantly activated miR-203 promoter-driven luciferase activity (Fig. 2A). Furthermore, the effect of IRF1 overexpression on miR-203 expression was analyzed by miRNA qRT-PCR. As shown in Fig. 2B, IRF1 overexpression increased endogenous mature miR-203 expression 8-fold compared with the control vector. These results indicate that miR-203 transcription is regulated by IRF1.

The interaction between IRF1 and the miR-203 promoter was further confirmed by ChIP qRT-PCR assays. As shown in Fig. 2C, the *in vivo* binding of IRF1 to the miR-203 promoter was significantly higher than that of the control. To identify IRF1 binding sites in the miR-203 promoter sequence, we mutated the predicted IRF1 binding sites (Fig. 1C). As shown in Fig. 2D, mutation of IRF1 binding site 2 (pGL3-mut 2) abolished the effects of IRF1 on the reporter, while mutating binding site 1 had no effect. According to these results, IRF1 regulates the expression of miR-203 by binding to site 2 (CACTT).

### MiR-203 suppresses *BANF1* expression by targeting the 3’UTR of *BANF1*

**Fig 3:**
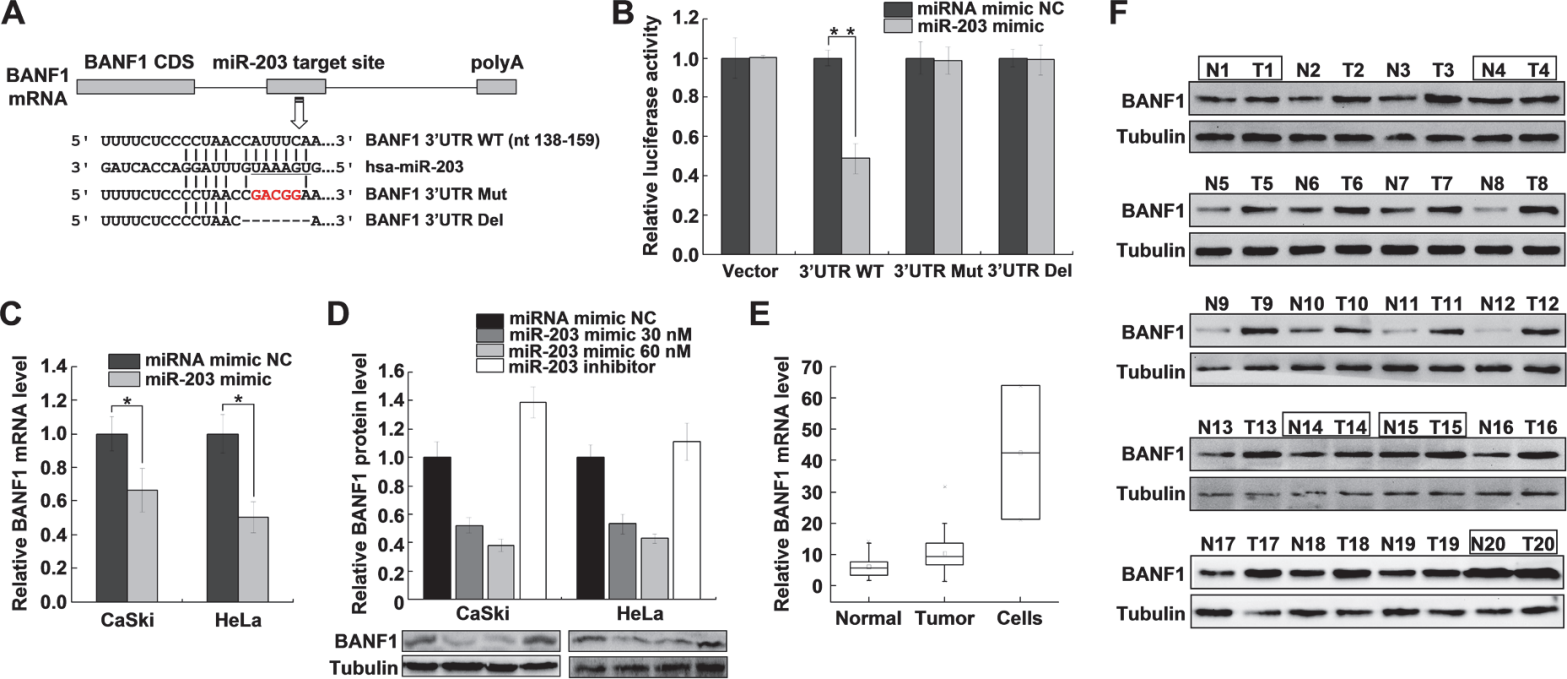
MiR-203 suppresses *BANF1* expression by targeting the *BANF1* 3’UTR. (A) Mature miR-203 sequences and recognition sites within the 3’UTR of *BANF1*. The seed sequence of miR-203 is underlined. The wild type (WT), mutated (Mut) and deleted (Del) *BANF1* 3’UTR recognition sites are also shown. (B) The relative luciferase activity of constructs containing the *BANF1* WT, Mut or Del 3’UTRs. Luciferase constructs were co-transfected with miRNA mimic negative control (NC, 30 nM) or miR-203 mimic (30 nM) into HeLa cells. Renilla luciferase activity was measured 36 h after incubation and normalized to firefly luciferase activity. An asterisk indicates significant downregulation of pre-miR-203 compared with the expression of the WT *BANF1* 3’UTR construct. Data are representative of three independent experiments. (C) QRT-PCR analysis of *BANF1* mRNA from CaSki and HeLa cells transfected with miRNA mimic NC (30 nM) or miR-203 mimic (30 nM). Data were normalized to the level of GAPDH mRNA, and the ratio of *BANF1*/GAPDH in the negative control was set to 1. Data are representative of three independent experiments. (D) Western blot analysis of endogenous *BANF1* expression in CaSki and HeLa cells 48 h after transfection with miR-203 mimic or inhibitor. Tubulin served as a loading control. The results are representative of three independent experiments. (E) *BANF1* expression levels in human normal cervical tissues, cervical cancer tissues and cervical cancer cell lines (CaSki and HeLa) were measured by qRT-PCR, using GAPDH as an internal control. (F) Western blot analysis of BANF1 levels in 20 paired tumor tissues (T) and adjacent normal cervical tissues (N). Tubulin served as a loading control. The five paired samples that exhibited no differences in BANF1 expression are indicated by boxes. *P<0.05, **P<0.01.

To explore the function of miR-203, we searched for potential miR-203 target genes using miRNA databases (TargetScan, miRanda, and miRecords). As shown in Fig. 3A, the seed region of miR-203 is perfectly complementary to the target sequence in the 3’UTR of *BANF1*. To validate this in silico prediction, we cloned the portion of the *BANF1* 3’UTR containing the miR-203 target site into the luciferase reporter plasmid psiCHECK-2. We then performed a luciferase reporter assay following co-transfection of the reporter constructs with the miR-203 mimic into HeLa cells. In the presence of the miR-203 mimic, the luciferase activity of the reporter construct containing the *BANF1* 3’UTR was significantly reduced, whereas that of the unmodified construct was not changed (Fig. 3B). Furthermore, mutation (Mut) and deletion (Del) of the miR-203 seed sequence both abrogated the miR-203-mediated reduction in luciferase activity (Fig. 3B). *BANF1* mRNA levels were measured by qRT-PCR, which showed decreased levels of *BANF1* mRNA in the presence of the miR-203 mimic (Fig. 3C). To further confirm that *BANF1* is a target of miR-203, we examined the endogenous *BANF1* protein level via western blotting after transiently transfecting the miR-203 mimic and inhibitor into HeLa and CaSki cells. As shown in Fig. 3D, the *BANF1* expression level decreased as the concentration of transfected miR-203 mimic was increased, while the *BANF1* expression level increased when cells were transfected with the miR-203 inhibitor. These results indicate that miR-203 can directly target the *BANF1* 3’UTR and downregulate *BANF1* expression.

To determine whether reduced miR-203 expression is correlated with the *BANF1* expression levels in cervical cancer, we evaluated the *BANF1* levels in 20 paired cervical cancer tissue samples and their adjacent normal cervical samples as well as in cervical cancer cell lines (HPV 16+ CaSki and HPV 18+ HeLa) via qRT-PCR. *BANF1* expression was significantly higher in cervical cancer cells and tissues than in normal tissues (Fig. 3E). In addition, western blot analysis showed that expressions of BANF1 was elevated in 15 out of 20 paired (75%) tumor tissues (T) than in normal (N) cervical tissues (Fig. 3F). The other 5 paired samples exhibited no differences in BANF1 expression are indicated by boxes. These results suggest that the abnormal decrease in the miR-203 levels in cervical cancer is related to the high expression of *BANF1*.

### IRF1 regulates miR-203 and indirectly influences the expression of *BANF1*

**Fig 4:**
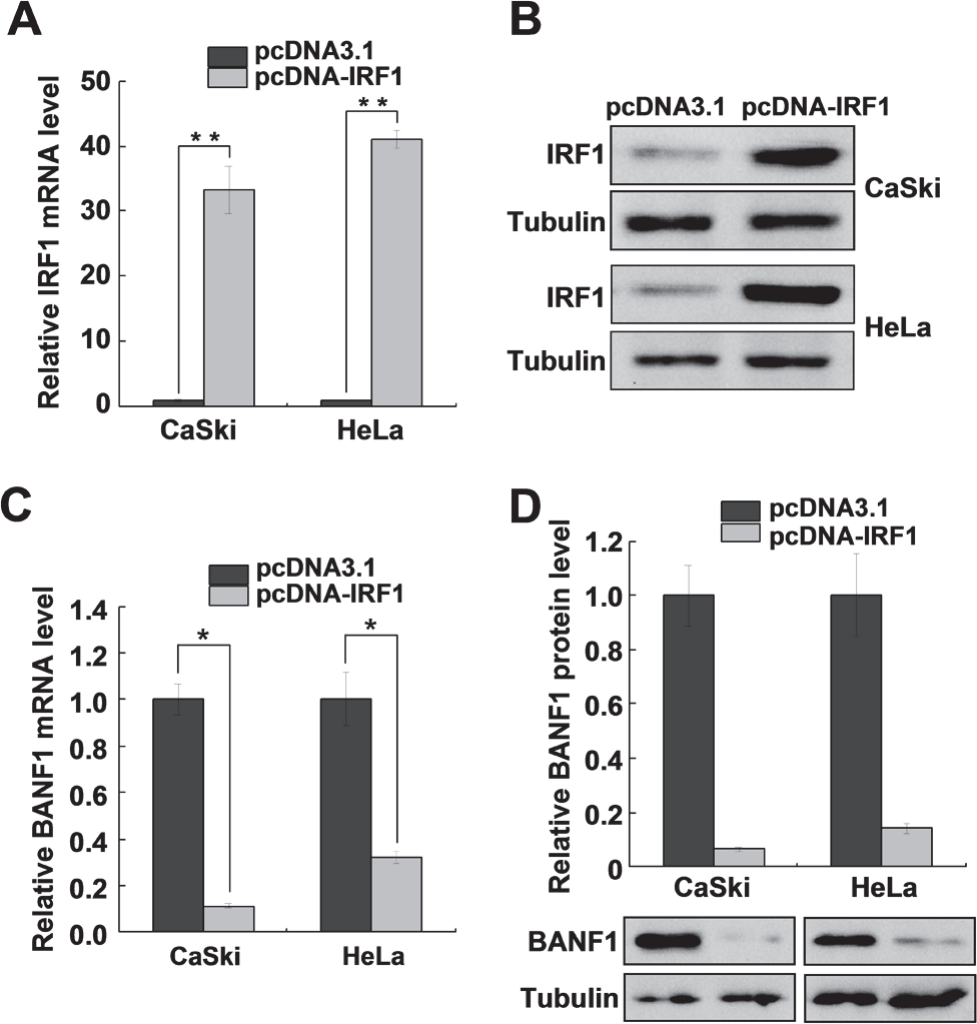
IRF1 indirectly regulates *BANF1* expression. (A, B) QRT-PCR and western blot analysis of IRF1 mRNA and protein levels in CaSki and HeLa cells transfected with pcDNA-IRF1. IRF1 mRNA and protein levels were significantly increased when transfected with pcDNA-IRF1. (C) QRT-PCR analysis of *BANF1* mRNA expression in HeLa cells transiently transfected with control vector or pcDNA-IRF1. The data were normalized to GAPDH expression. (D) Western blot analysis of BANF1 expression in CaSki and HeLa cells transiently transfected with pcDNA-IRF1. The results are representative of three independent experiments. *P<0.05.

To validate whether IRF1 regulation of miR-203 can mediate changes in *BANF1* expression, we evaluated *BANF1* expression by qRT-PCR and western blotting under IRF1 overexpression. As shown in Fig. 4A and B, IRF1 mRNA and protein levels increased significantly in CaSki and HeLa cells after cells were transfected with pcDNA-IRF1. Interestingly, *BANF1* mRNA and protein levels were reduced in CaSki and HeLa cells transiently transfected with pcDNA-IRF1 (Fig. 4C and 4D), which strongly suggesting that IRF1 stimulates miR-203 expression, which subsequently downregulates *BANF1* expression.

### MiR-203 suppresses cervical cancer cell proliferation and colony formation

**Fig 5:**
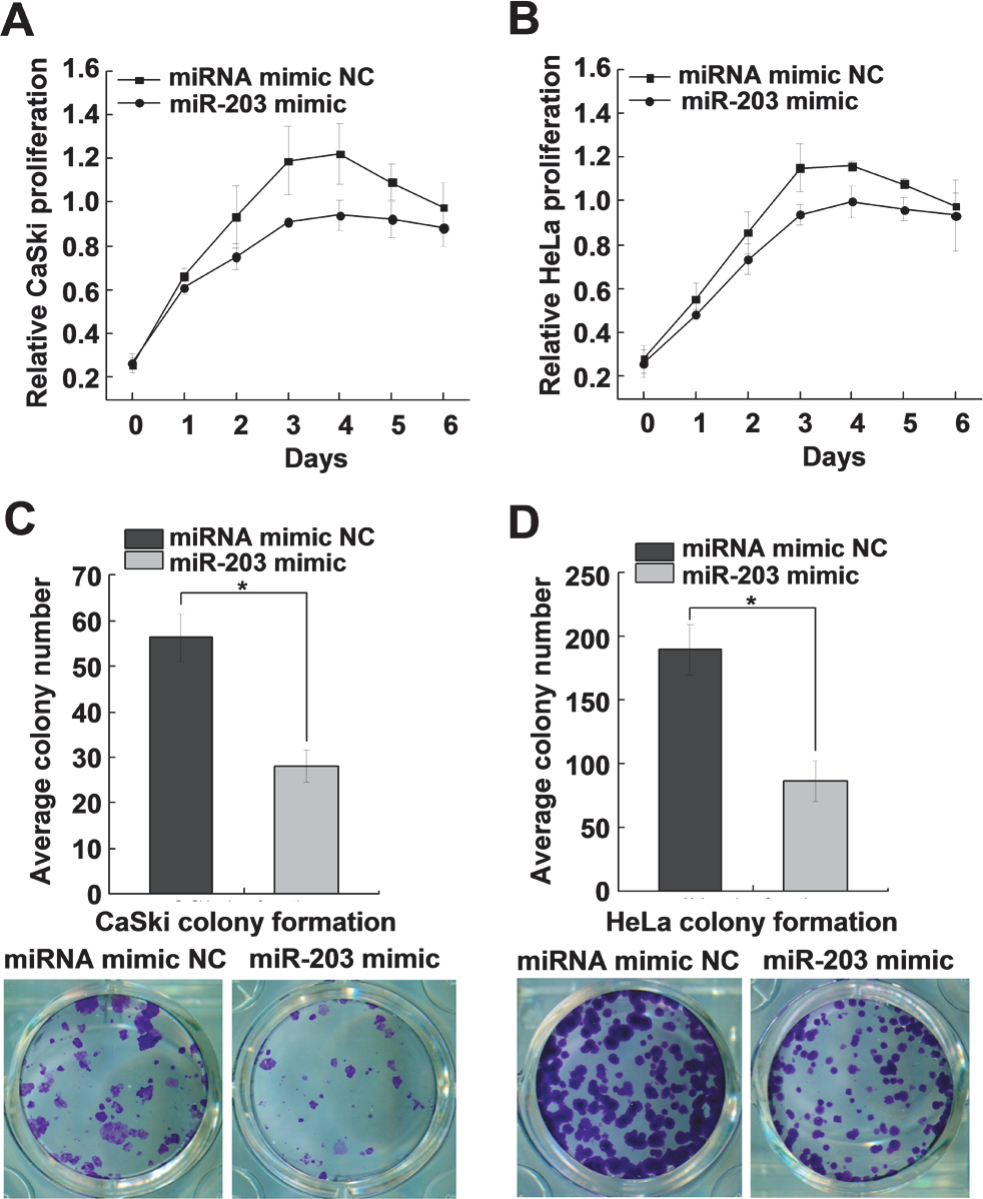
MiR-203 suppresses cervical cancer cell proliferation and colony formation. (A, B) Cell proliferation assays of CaSki and HeLa cells transfected with miR-203 mimic or negative control. (C, D) Colony formation assays of CaSki and HeLa cells transfected with miR-203 mimic or negative control. All data are representative of three independent experiments. *P<0.05.

We explored the cellular functions of miR-203 in cervical cancer and found that miR-203 overexpression markedly suppressed the growth of CaSki and HeLa cells (Fig. 5A and B). Similarly, colony formation capacity was decreased in CaSki and HeLa cells when miR-203 was over expressed (Fig. 5C and D).

### MiR-203 is involved in cell cycle regulation and suppresses cervical cancer cell migration and invasion *in vitro*

**Fig 6:**
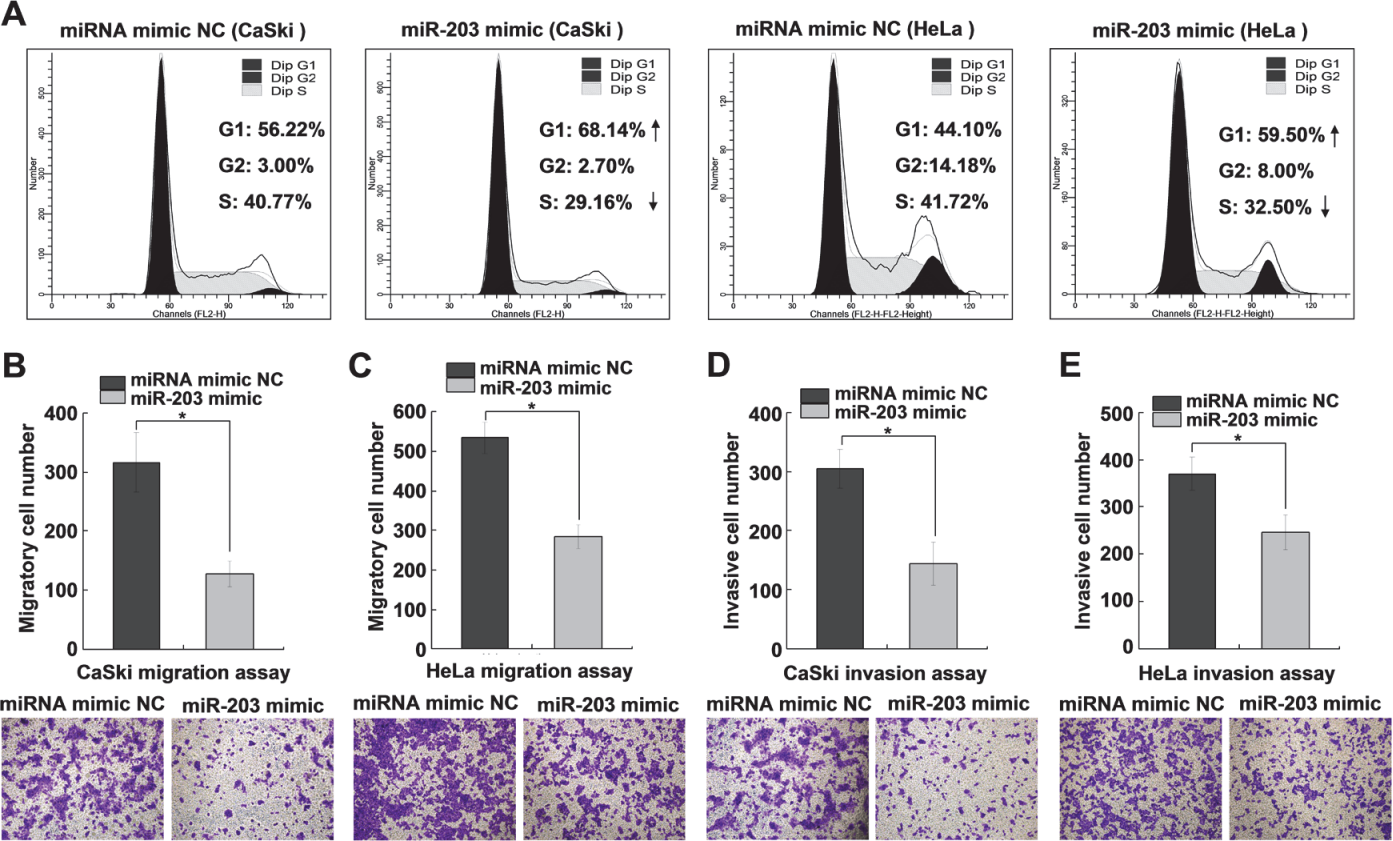
MiR-203 is involved in cell cycle regulation and suppresses cervical cancer cell migration and invasion. (A) Flow cytometry analysis showing the effect of miR-203 overexpression on the cell cycle of CaSki and HeLa cells. Cells were transfected with 30 nM miR-203 mimic or negative control (NC) and collected, stained and analyzed 48 h after transfection. (B, C) Transwell migration assays of CaSki and HeLa cells transfected with miR-203 mimic or NC. The migrated cells were quantified and representative images are shown at the bottom. (D, E) Transwell invasion assays of CaSki and HeLa cells transfected with miR-203 mimic or NC. The invaded cells were quantified and representative images are shown. The results are representative of three independent experiments. *P<0.05.

Cells transfected with the miR-203 mimic were subjected to cell cycle analysis via flow cytometry. As shown in Fig. 6A, cervical cancer cells overexpressing miR-203 had a higher percentage of G1-phase cells and a lower percentage of S-phase cells, suggesting that miR-203 induces G1 arrest. In addition, transwell assays with or without Matrigel showed that miR-203 overexpression in CaSki and HeLa cells suppressed the migration and invasion ability compared with the negative control (NC) cells (Fig. 6B, C, D and E).

### *BANF1* knockdown suppresses cell colony formation, migration and invasion

**Fig 7:**
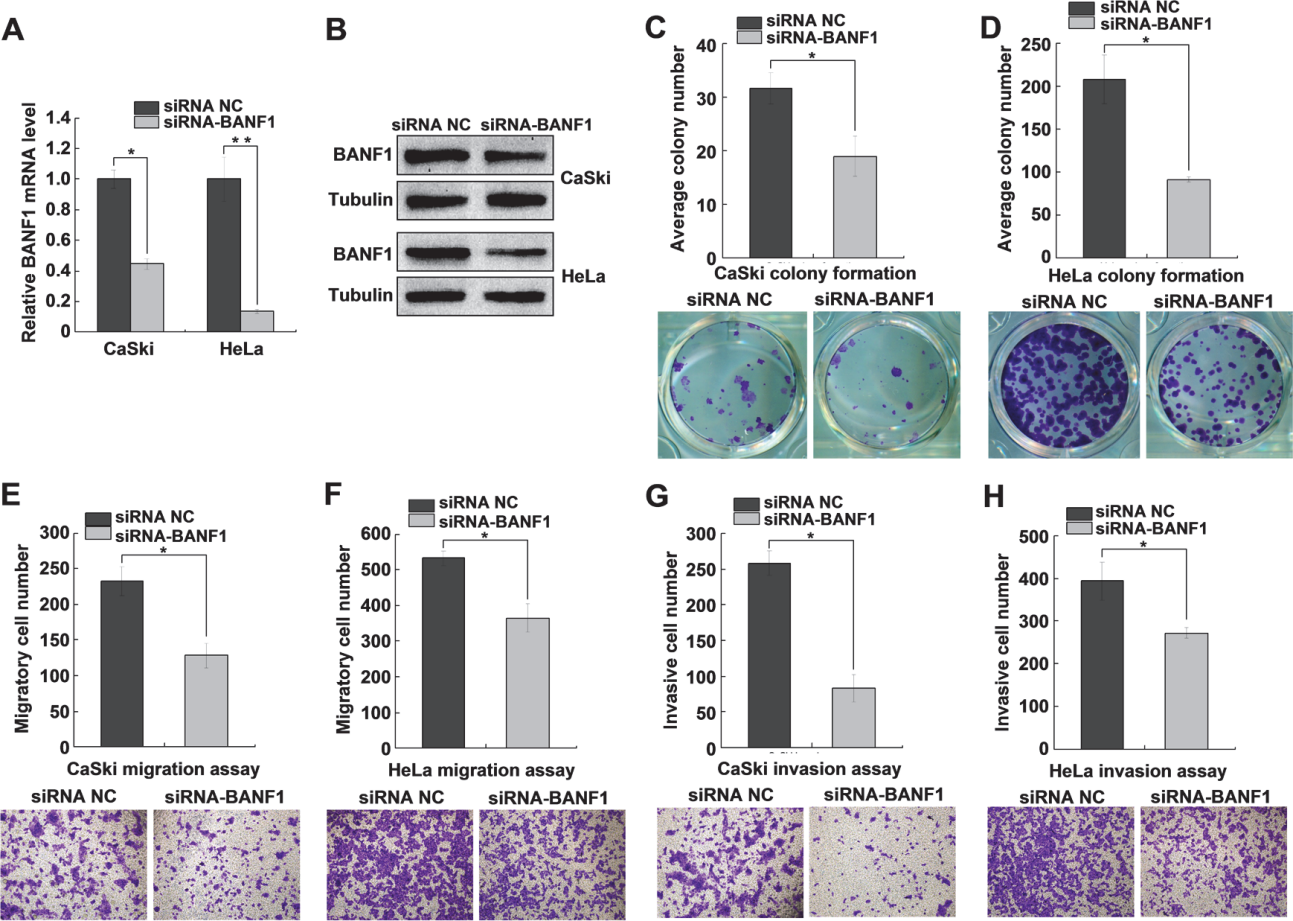
*BANF1* knockdown suppresses cell colony formation, migration and invasion. (A, B) QRT-PCR and western blot analysis of *BANF1* levels in *BANF1*-knockdown CaSki and HeLa cells. *BANF1* mRNA and protein levels were significantly reduced when transfected with siRNA-BANF1. (C, D) Colony formation assays revealed that the average colony formation number was reduced in *BANF1*-knockdown CaSki and HeLa cells. (E, F) Cell migration assays revealing that the migrated cells were decreased by knocking down *BANF1*. (G, H) Cell invasion assays revealing that the invaded cells were decreased by knocking down *BANF1*. All data are representative of three independent experiments. *P<0.05.

To further validate whether *BANF1* is involved in miR-203 mediated colony formation, migration and invasion, siRNA targeting *BANF1* was designed and transfected into CaSki and HeLa cells. The results showed that siRNA targeting *BANF1* significantly decreased *BANF1* mRNA and protein levels in CaSki and HeLa cells (Fig. 7A and B). As expected, knockdown of endogenous *BANF1* by siRNA resulted in a significant decrease in colony formation (Fig. 7C and D). Similarly, the migration and invasion abilities of CaSki and HeLa cells were also significantly decreased by the *BANF1* siRNA but not by the negative control (NC) siRNA (Fig. 7E, F, G and H).

## Discussion

A growing number of studies have shown that miRNAs are involved in various physiological processes and are associated with the development of diseases through their ability to regulate target genes [22,23]. Regulation of miRNA expression is important for maintaining cellular homeostasis; however, the molecular mechanisms regulating miRNA gene transcription are not well understood [24,25]. Regulation of expression at the transcriptional level is a critical step in miRNA biosynthesis [26].

miRNA genes are transcribed as a unit or cluster and can reside in either intergenic regions or within the introns or exons of coding genes [27]. When miRNAs reside in intragenic regions, they can be transcribed independently if they have their own promoters [27], or they can be transcribed together with the host gene if they lack their own promoters. Identification of TSSs and promoters is critical for understanding the mechanisms and transcription factors that mediate miRNA expression [28]. The miR-203 gene is localized in an intergenic region and is transcribed independently. Our study identified miR-203 TSSs by 5’RACE and subsequently identified the miR-203 promoter sequence.

Having multiple TSSs is an important mechanism for increasing gene complexity and contributing to functional diversity, a phenomenon that has been well studied for protein-coding genes. In complex species, it is common for genes to have several different TSSs that may be active under different conditions [29]. It has been reported that the human miRNA gene cluster miR-23a-miR-27a-miR-24-2 and *Drosophila melanogaster* miRNAs miR-276a and miR-277 have alternative TSSs [30]. In this study, we identified two TSSs for miR-203. In most cases, the variable transcripts for each miRNA may produce the same mature miRNA [31]. Therefore, forming multiple transcripts has little influence on the function of a miRNA. However, we speculate that distinct TSSs of miRNAs might have different transcription efficacies and could regulate the expression level of the miRNAs. Further understanding of alternative splicing may provide more information on miRNA gene regulation.

**Fig 8:**
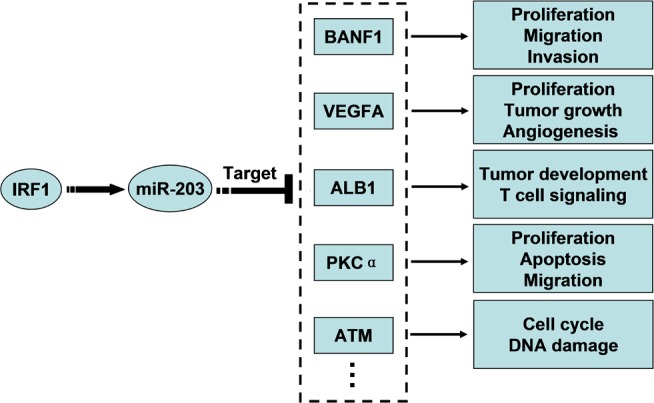
Model for the potential roles of IRF1 and miR-203 in diverse biological processes.

Our study establishes a linear signaling pathway (*IRF1* to miR-203 to *BANF1*) (Fig. 8). *IRF1* encodes interferon regulatory factor 1, a member of the interferon regulatory transcription factor (IRF) family. IRF1 is decreased in cancer tissues and functions in the immune response, apoptosis regulation, DNA damage repair and tumor suppression [32–34]. Previous studies have validated that miR-203 regulates a series of target genes, including *VEGFA* [18], *ALB1* [35], *PKC*α [16], and *ATM* [36] (Fig. 8). These target genes are involved in biological processes similar to IRF1. In addition, previous studies have shown that HPV E7 is functionally associated with IRF1. HPV E7 interferes with the transactivation function of IRF1 by recruiting histone deacetylases to the *IRF1* promoter [37,38]. The low expression of IRF1 in cervical cancer may contribute to the decreased levels of miR-203 by blocking the transcription of miR-203.

The protein product of the miR-203 target gene, *BANF1* Barrier-to-autointegration factor, is a conserved human chromatin protein that is mainly involved in nuclear assembly and chromatin decondensation [39,40] and is exploited by retroviruses. BANF1 binds double-stranded DNA non-specifically and is a host component of pre-integration complexes [41,42]. HPV integration into the human genome is a critical step for HPV infection and cervical cancer formation [43]. Therefore, increased *BANF1* expression in cervical tissue may contribute to the integration of HPV.

In summary, miRNAs play important roles in normal tissues, and their study provides a more comprehensive understanding of cancer development. The detection of aberrant miRNA expression in tumor tissues may be useful as a novel molecular biomarker for cancer. Because miRNA expression can be regulated by several factors, including epigenetic modifications, transcription factor regulation, and genetic alterations, the mechanisms involved in miRNA deregulation are a matter of intense research. Further study of miRNAs will result in a more comprehensive understanding of the mechanisms of tumor development and provide methods and a theoretical basis for clinical treatment.

## Supporting Information

S1 Table Primer sequences used in this study. (DOC)
