# Correction: *BANF1* Is Downregulated by IRF1-Regulated MicroRNA-203 in Cervical Cancer

**DOI:** 10.1371/journal.pone.0129756

**Published:** 2015-06-01

**Authors:** The PLOS ONE Staff

The Funding section was unintentionally omitted. The complete, correct funding information is:

This work was supported by the National Natural Science Foundation of China (91129730, 21132004 to H. L and 31200988 to W. M), Specialized Research Fund for the Doctoral Program of High Education (20110071130007), National Program on the Key Basic Research Project (973 Program, 2009CB825601), Shanghai Science and Technology Commission (13DZ2252000). The funders had no role in study design, data collection and analysis, decision to publish, or preparation of the manuscript.

The publisher apologizes for the error.

## References

[1] Mao L, Zhang Y, Mo W, Yu Y, Lu H (2015) *BANF1* Is Downregulated by IRF1-Regulated MicroRNA-203 in Cervical Cancer. PLoS ONE 10(2): e0117035 doi:10.1371/journal.pone.0117035 25658920 10.1371/journal.pone.0117035 PMC4319761

