# Supplementary material for: BANF1 Is Downregulated by IRF1-Regulated MicroRNA-203 in Cervical Cancer
Source: PLoS One. 2015 Feb 6;10(2):e0117035. doi: 10.1371/journal.pone.0117035 (PMC4319761; doi:10.1371/journal.pone.0117035)
Supplement: S1 Table — (DOC) [file pone.0117035.s001.doc]

Table S1. Primer sequences used in this study

| primer name | primer sequences |
| --- | --- |
| RT-miR-203 | GTCGTATCCAGTGCAGGGTCCGAGGTATTCGCACTGGATACGACctagtg |
| RT-RNU6B | GTCGTATCCAGTGCAGGGTCCGAGGTATTCGCACTGGATACGACAAAAATAT |
| qRT-RNU6B-F | TTCCTCCGCAAGGATGACACGC |
| qRT-miR-203-F | gccgcgGTGAAATGTTTAGGAC |
| qRT-miRNA-R | GTGCAGGGTCCGAGGT |
| BANF1 3’UTR-F-XhoI | ccgctcgagGGTGCTAAAGAAAGATGAA |
| BANF1 3’UTR-R-NotI | ataagaatgcggccgcTTTTGAAAGCCACTGACT |
| BANF1 3’UTR-mut-F | CTCCCCTAACCGACGGAACTTTTTTTTGGA |
| BANF1 3’UTR-mut-R | TCCAAAAAAAAGTTCCGTCGGTTAGGGGAG |
| BANF1 3’UTR-del-F | GTTTTCTCCCCTAACACTTTTTTTTGGA |
| BANF1 3’UTR-del-R | TCCAAAAAAAAGTGTTAGGGGAGAAAAC |
| qRT-BANF1-F | CCAGCCCTCATCCAGAGT |
| qRT-BANF1-R | GCAAGAGCGAGAATCCAA |
| qRT-GAPDH-F | ATGGGGAAGGTGAAGGTCG |
| qRT-GAPDH-R | CTGGAAGATGGTGATGGGA |
| miR-203 5’RACE outer | CGGGTCTAGTGGTCCTAAACATT |
| miR-203 5’RACE inner | AACTGTTAAGAACCACTGGACCC |
| miR-203 promoter-F-XhoI | ccgctcgagATGGCTCCAGACTTGGGGCA |
| miR-203 promoter-R-HindIII | cccaagcttCGACTGATCCTCCACGGC |
| IRF1-F-XhoI | ccgctcgagCCACCATGCCCATCACTCGGAT |
| IRF1-R-HindIII | cccaagcttCTACGGTGCACAGGGAAT |
| qRT-IRF1-F | AGCCCTGATACCTTCTCTGAT |
| qRT-IRF1-R | ATGTGCCAGTCGGGGAGAGT |
| ChIP qRT-F | GCGTGGGAAATGAGGAGG |
| ChIP qRT-R | GGGAAGGGGAGGGGTAAA |
| IRF1-miR-203-mut 1-F | CTCCCTCCAGCCCTGCTTTCTCTTTA |
| IRF1-miR-203-mut 1-R | TAAAGAGAAAGCAGGGCTGGAGGGAG |
| IRF1-miR-203-mut 2-F | CCTCCAGTTTAGAGGTCTCTTTACC |
| IRF1-miR-203-mut 2-R | GGTAAAGAGACCTCTAAACTGGAGG |

Mutant sequences were indicated in red.
